# Biotechnological uses of purified and characterized alkaline cellulase from extremophilic *Bacillus pumilus* VLC7 from Lake Van

**DOI:** 10.1007/s11274-025-04271-4

**Published:** 2025-02-04

**Authors:** Aysun Ayse Yilmaz, Sumeyra Gurkok

**Affiliations:** 1 Institute of Natural and Applied Sciences, Atatürk University, Erzurum, Turkey; 2 Department of Biology, Science Faculty, Ataturk University, Erzurum, Turkey

**Keywords:** Alkaline cellulase, *Bacillus pumilus*, Biopolishing, Dye removal, Lake Van

## Abstract

The present study focuses on the isolation and characterization of a bacterium from Lake Van adapted to an alkaline pH environment with significant cellulase production potential. The identified isolate, *Bacillus pumilus* VLC7 (GenBank Acc No: OR415888.1), exhibited the highest cellulase activity among other alkaliphilic isolates. The purification employing protein precipitation by ammonium sulfate, ultrafiltration, and ion exchange chromatography resulted in 20-fold purification with a specific activity of 16 U/mg protein. Cellulase had a molecular weight of 76 kDa, 3.13 mM K_m_ and 0.160 U/mg V_max_ values. The enzyme displayed maximum activity at pH 9.0 and 40 °C and retained at least 80% of its activity at temperatures of 25–60 °C for 90 min and pH 4–12 for one hour. Stability tests also revealed the enzyme’s resilience to various reagents, metal ions, organic solvents, and detergents. Furthermore, the biotechnological applications of cellulase were explored, demonstrating its effectiveness in fabric biopolishing (removing pilling), as well as in the removal of fabric dyes. The research findings underscore the potential of *B. pumilus* VLC7 as a valuable source for eco-friendly industrial biotechnology applications.

**Graphical Abstract:**

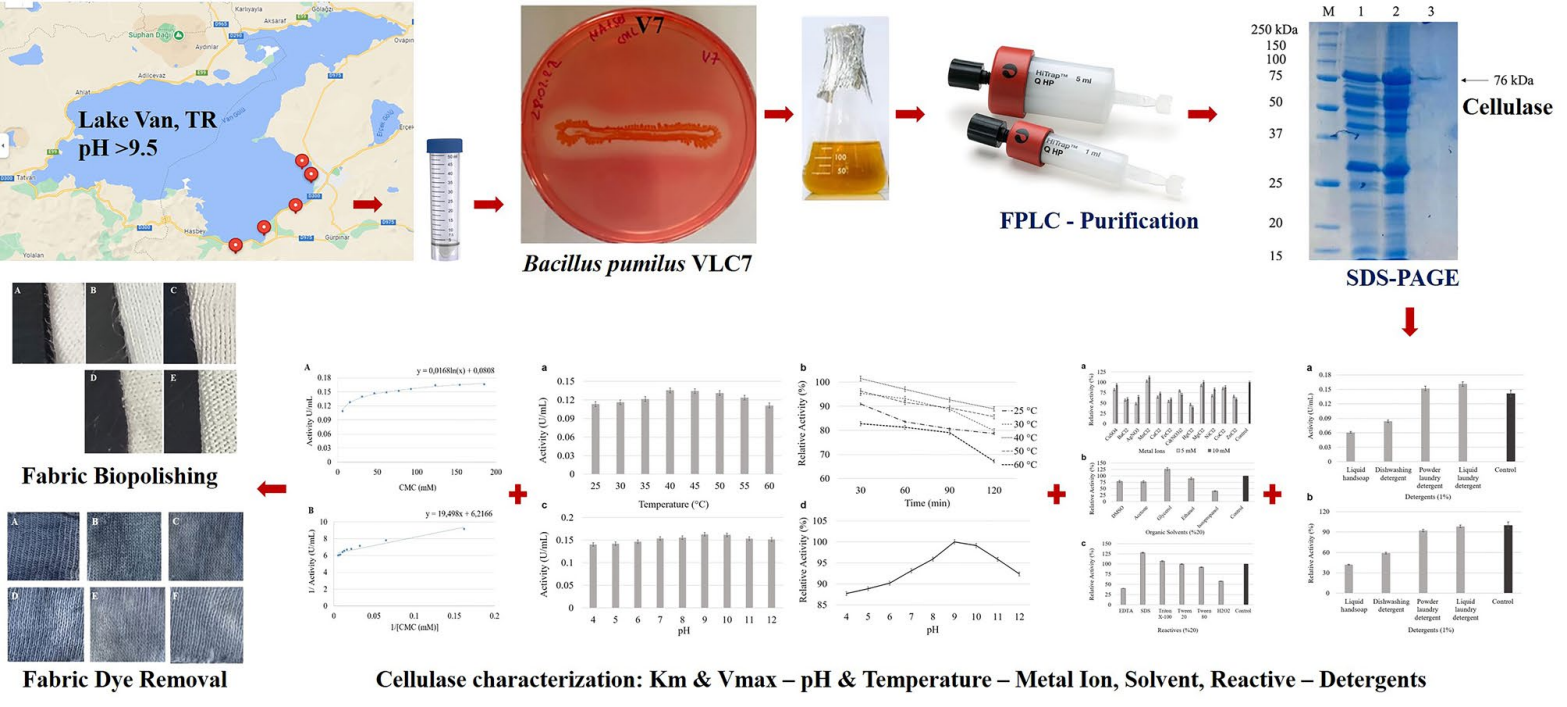

**Supplementary Information:**

The online version contains supplementary material available at 10.1007/s11274-025-04271-4.

## Introduction

Cellulases are enzymes that break down recalcitrant cellulose and hemicellulose fractions into fermentable sugars and have emerged as invaluable assets in biotechnological applications. Through enzymatic hydrolysis, cellulases pave the way for biofuel and bio-based chemical production and also offer a potent tool for waste valorization and environmental remediation, enabling the degradation of lignocellulosic waste streams and organic pollutants (Gurkok et al. 2024; John and Selvarajan 2024; Sutaoney et al. 2024; Kant et al. 2024). Cellulases also find extensive utility in the textile industry, where they catalyze biopolishing reactions, effectively removing surface fuzz and pilling from fabrics to impart a smoother texture and enhanced aesthetic appeal (Kant et al. 2024; Msakni et al. 2024; Dil et al. 2024). Furthermore, they facilitate enzymatic desizing and dye removal, fostering resource-efficient textile processing.

Microorganisms constitute the reservoir of industrial cellulolytic enzymes. The heightened catalytic activity, enhanced stability, rapid growth rates, and metabolic versatility of microorganisms underline a sustainable approach to cellulase production. Moreover, microbial cellulases offer scalability, cost-effectiveness, minimal by-product formation, and operational efficiency, rendering them indispensable components of industrial bioprocesses. Therefore, the quest for efficient cellulase-producing microorganisms assumes paramount importance in various biotechnological applications. Among bacteria, cellulase production is notably prevalent within the *Bacillus* genus (Ariffin et al. 2006; Asha and Sakthivel 2014; Gaur and Tiwari 2015; Balla et al. 2022). *Bacillus* species are renowned for their adaptability to diverse environmental conditions, metabolic versatility, and robust enzyme secretion systems, making them attractive candidates for cellulase production and underscores their significance as key players in biotechnological endeavors.

In the pursuit of identifying a robust alkaliphilic cellulase producer for use in the textile and detergent industries, the selection of Lake Van as the sampling locale represents a strategic choice imbued with scientific rationale. It was a deliberate choice to uncover microbial reservoirs harboring exceptional enzymatic potential adapted to alkaline environments. Lake Van, the world’s largest soda lake, is characterized by its unique physicochemical properties and alkaline milieu. It is a saline lake with a distinct soda chemistry, exhibiting a pH of 9.52 and a salt concentration of 0.224% (Çiftçi et al. 2008). The waters of Lake Van offer a fertile landscape for the proliferation of specialized microorganisms endowed with enzymatic machinery tailored to thrive in alkaline settings.

Drawing inspiration from the extreme environments of Lake Van, extremophilic bacteria adapted to alkaline pH regimes were meticulously screened. An efficient cellulase-producing isolate emerged and was primed for further exploration. Employing a suite of purification techniques including protein precipitation, ultrafiltration, and chromatography, the cellulase was purified for in-depth characterization. Comprehensive analyses unveiled pivotal insights into the purified enzyme’s molecular properties, kinetic constants, pH and temperature optima, as well as its resilience in diverse chemical milieus. Beyond fundamental characterization, the study explored practical applications of cellulases across industrial domains, notably in detergent, and textile industries.

## Materials and methods

### Isolating alkaline-adapted bacteria

Three water samples from five different locations of Lake Van, Turkey, (38.520374, 43.314816; 38.309119, 43.040275; 38.560118, 43.282007; 38.427571, 43.258307; 38.422899, 43.285634) were collected and inoculated on nutrient agar (NA), pH 9 at 30 °C. Pure cultures of the isolates were kept at -87 °C in 20% glycerol stocks.

### Assessment of cellulase activity among alkaliphilic strains

To assess cellulase activity in isolates, pure cultures were introduced into carboxymethyl cellulose - agar (CMC-A) medium (NA with 10 g/L carboxymethyl cellulose), pH 9, and maintained at 30 °C for 48–72 h. Subsequently, colonies were treated with 0.1% Congo red for 15 min. Following staining, the solution was decanted, and a 1 M NaCl solution was applied for a 30-minute wash cycle. The diameters of cellulolytic zones surrounding the isolates were then gauged and those forming the most expansive zones (Gohel et al. 2014).

### Cultivation conditions for cellulase production

The most promising cellulolytic isolates were cultured in 100 mL of carboxymethyl cellulose - broth (CMC-B) composed of 10 g/L CMC, 0.2 g/L MgSO_4_.7H_2_O, 5 g/L NaCl, 10 g/L peptone, 2 g/L yeast extract, 1 g/L K_2_HPO_4_, and 0.1 g/L CaCl_2_ (adjusted to pH 9). The medium composition was modified based on Aygan and Arikan (2009). Cultures were incubated at 30 °C and 180 rpm for 5 days, and the activities of the cultures were monitored.

### Measurement of cellulase activity

Post-incubation, the cultures were centrifuged at 4 °C and 6000 rpm for 10 min to separate the supernatant, which was used as crude enzyme. Freshly prepared 1% CMC solution in 50 mM Glycine-NaOH buffer at pH 9 (referred to as Buffer A) was used as the substrate solution. Both 0.5 mL substrate solution and 0.5 mL crude enzyme were homogenized and kept in a 35 °C water bath for 30 min. Following incubation, 1 mL of 3,5- Dinitrosalicylic acid (DNS) reagent (Aktas et al. 2023) was added, and the mixture was boiled for 5 min to stop the reaction. After cooling to room temperature, absorbance at 540 nm was measured (Miller 1959), and carboxymethyl cellulase activity was calculated based on the glucose standard graph. One unit of cellulase enzyme activity (1 U) was evaluated as the amount of enzyme that produced 1 µmol glucose in 1 min under the test conditions.

To determine exo-β-1,4-glucanase activity and endo-β-1,4-glucanase activity, 5 mM *p*-nitrophenyl β-*D*-cellobioside (*p*NPC) and 5 mM *p*-nitrophenyl β-D-glucopyranoside (*p*NPG) substrate solutions prepared in Buffer A were used, respectively. 100 µL of crude enzyme and 900 µL of respective substrate solutions were mixed and incubated at 35 °C for 30 min. The reactions were terminated by adding 100 µL of Na₂CO₃. Absorbance measurements were performed at 410 nm. As a control, 100 µL Buffer A was used instead of the crude enzyme. Exo-β-1,4-glucanase and β-1,4-glucanase activity calculations were made using a *p*-nitrophenol standard curve. All the enzymatic experiments were conducted in triplicate.

### Identification of cellulase-producing isolates

The bacterial isolate demonstrating the highest cellulase activity, as determined by qualitative and quantitative cellulase activity assays, was selected. The isolate was identified utilizing 16 S rDNA sequence analysis conducted by Macrogen, South Korea and classical identification techniques. The isolate’s morphological characteristics (colony color and shape, cell shape, Gram staining, and KOH test) and biochemical properties (oxidase, amylase, protease, hemolysis, H₂O₂ catalase, urease, lipase, asparaginase, glutaminase, maltose utilization, etc.) were determined. Subsequently, the derived sequence data was deposited into the National Center for Biotechnology Information (NCBI) database, and GenBank accession number was obtained.

The phylogenetic tree was constructes using the Phylogeny.fr platform (https://www.phylogeny.fr/phylogeny.cgi), which integrates alignment and phylogeny programs. Multiple sequence alignment was carried out using the NCBI MSA Viewer 1.25.0. On the Phylogeny.fr platform, tree construction was completed using the maximum-likelihood method with the default settings (Kuhner and Felsenstein 1994).

### Extraction and purification of cellulase

At the end of the 96 h of incubation in CMC-B, bacterial cells were removed by centrifugation at 6000 rpm for 25 min at 4 °C. After centrifugation, ammonium sulfate was gradually added to the supernatant to achieve final concentrations of 20%, 40%, and 60%, respectively, and protein precipitation was carried out. For each concentration, ammonium sulfate was added over 30 min, and the mixture was stirred at 4 °C for a minimum of 4 h. Following this, the solution was centrifuged at 10,000 rpm for 30 min to precipitate the proteins, and the supernatant was separated to continue precipitation at the next ammonium sulfate concentration. The precipitated proteins were dissolved in Buffer A. Ultrafiltration (Millipore, 10 kDa cut-off) was performed at 6000 rpm to remove the remaining ions and salts from the process and to concentrate the protein solution. The obtained samples were loaded onto an anion exchange 16/10 HiPrep Q XL column (Biorad, Biologic LP, USA) equilibrated using Buffer A. The samples were passed through the column at a flow rate of 1 mL/min using a NaCl gradient ranging from 0 to 1 M. Fractions were collected, and cellulase activity measurements were performed on each. Fractions showing high cellulase activity were pooled, concentrated by ultrafiltration, and stored at -20 ºC (Gurkok and Ozdal 2021; Hemsinli and Gurkok 2024) for subsequent enzyme characterization studies.

### Investigation into cellulase characteristics

#### Assessment of protein content and sodium dodecyl sulfate-polyacrylamide gel electrophoresis profiling

The assessment of protein content was carried out using the Lowry method, employing bovine serum albumin (BSA) as the standard for calibration (Lowry et al. 1951). Following quantification, sodium dodecyl sulfate-polyacrylamide gel electrophoresis (SDS-PAGE) was conducted to analyze the molecular weight distribution of the proteins. Samples of crude enzyme extract, ammonium sulfate precipitated protein, and purified cellulase (10 µg each) were loaded onto a 12% polyacrylamide gel and separated using the Bio-Rad Mini-PROTEAN^®^ Tetra Cell system (Laemmli 1970). Post-electrophoresis, the gel was stained with Coomassie Brilliant Blue R-250 for protein visualization. A molecular weight marker, Precision Plus Protein™ Unstained Standard (Bio-Rad), with a size range of 250–10 kDa, was used to estimate the molecular weights of the protein bands.

#### Determining km and vmax values for cellulase

The maximal velocity (V_max_) and the Michaelis-Menten constant (K_m_) for cellulase were determined using CMC as the substrate within a concentration range of 0.1–3% (Counotte and Prins 1979).

#### Evaluation of optimal temperature and pH for cellulase activity

To find the optimal temperature for the purified cellulase, enzyme activity assays were conducted across a range of temperatures from 25 to 60 °C, with all other conditions kept constant. Prior to the reaction, enzyme and substrate solutions were kept for 10 min at the specified temperatures.

To determine the optimum pH, the activity assays were conducted in a pH range of 4.0 to 12.0. The used buffers included 50 mM acetic acid-sodium acetate buffer for pH 4.0–5.0, potassium phosphate buffer for pH 6.0, tris-HCl buffer for pH 7.0–8.0, and glycine-NaOH buffer for pH 9.0–12.0. Additionally, the corresponding buffers were used to prepare the substrate solution to maintain the desired pH throughout the assay.

#### Assessment of enzyme durability across the temperature and pH variations

Temperature stability was assessed by incubating enzyme samples at a temperature range of 25–60 °C for periods of 30, 60, 90, and 120 min. The relative activity of the enzyme was calculated as a percentage of the activity observed in the unincubated control sample, which served as a reference for maximal enzyme activity.

To evaluate pH stability, enzyme solutions were incubated in buffers with a pH range of 6.0 to 12.0 for 1 h at 25 °C. Following this incubation, the relative activity was expressed as a percentage of the activity of the unincubated control sample. The control samples for temperature and pH stability assays were considered to be 100%.

#### Assessing the effects of metal ions, inhibitors, and solvents on cellulase activity

To examine the impact of metal ions, 5 mM and 10 mM solutions of various metal ions (AgNO₃, BaCl₂, CaCl₂, Cd(NO₃)₂, CoCl₂, CuSO₄, FeCl₂, HgCl₂, MgCl₂, MnCl₂, NiCl₂, and ZnCl₂) were prepared. Additionally, to investigate the effects of inhibitors, 20% solutions of various inhibitors such as EDTA (ethylenediaminetetraacetic acid), Triton X-100, H₂O₂, SDS, Tween 80, and Tween 20) were used. For evaluating the effects of solvents, 20% solutions of ethanol, DMSO (dimethyl sulfoxide), glycerol, acetone, and isopropanol were prepared. All solutions were dissolved in Buffer A. The enzyme and test solutions were incubated for 1 h at room temperature in a 1:1 ratio. Enzyme activity in the presence of each test solution was compared to that of a control sample, which was incubated under identical conditions without any test solutions, to calculate the relative activity.

#### Assessment of cellulase activity and stability in the presence of commercial detergents

To assess the impacts of commercial detergents on cellulase activity, standard activity assays were performed with the addition of 1% liquid soap, dishwashing detergent, liquid laundry detergent, and powdered laundry detergent. To evaluate the impacts of these detergents on cellulase stability, the enzyme was mixed with the detergent solutions (1% liquid soap, dishwashing detergent, liquid laundry detergent, and powdered laundry detergent in Buffer A) at a 1:1 ratio. The mixtures were incubated at room temperature for 1 h. Following incubation, cellulase activity was measured under standard conditions. The relative activity was then calculated by comparing it to a control sample, which was incubated under the same conditions but without detergents.

### Biotechnological applications of cellulase

#### Assessment of cellulase for surface fuzz removal from cotton fabrics

The depilling effect of cellulase on cotton fabric surfaces was evaluated by treating cotton fabric samples with cellulase-rich supernatant obtained from the bacterial culture grown in CMC-broth medium for 96 h. Fabric pieces of approximately 2–2.5 cm were immersed in 25 mL of enzyme solution, and the samples were subjected to shaking (100 rpm) to ensure uniform exposure for varying time intervals (30, 60, 90, and 120 min). After each incubation period, the treated fabric pieces were thoroughly rinsed with deionized water to remove any residual enzyme. The rinsed fabrics were subsequently dried at 60 °C for 1 h. The extent of fuzz removal from the fabric surface was evaluated through visual inspection, providing qualitative insights into the efficiency of cellulase in reducing pilling (Duran et al. 2008).

#### Exploring the potential of cellulase in textile dye removal: a case study with indigo

To evaluate cellulase’s ability to degrade fabric dyes, indigo dye was used as the dyeing agent. Indigo, which is insoluble in water, was converted to its soluble form, leuco-indigo, under alkaline conditions (Pathak and Madamwar 2010). Soluble leuco-indigo was prepared by reducing indigo dye powder (5 g/L) with NaOH (2 g/L) and Na_2_S_2_O_4_ (2 g/L). Fabric pieces, measuring 2–2.5 cm, were added to the dye solution. Dyeing was performed at room temperature for 1 h. After dyeing, the samples were air-oxidized, rinsed, and air-dried. Fabric pieces were immersed in 5 mL of enzyme solution for 1, 3, 5, 15, and 20 h. After incubation, the fabric pieces were washed with distilled water to remove any residual dye. The cleaned fabrics were dried in an oven at 60 °C for 1 h, and the dye concentration levels on the fabric were visually inspected (Tayade and Adivarekar 2014).

### Statistical data analysis

All experiments were carried out in triplicate, and the resulting data were analyzed using Microsoft Excel 2016 (Microsoft Corporation). The data are reported as mean values with standard deviations. Statistical significance was assessed at a 95% confidence level, with *p*-values less than 0.05 (*p* < 0.05) considered significant.

## Results

### Isolation and screening of alkaliphilic bacteria from Lake Van for cellulase production

Alkaliphilic isolates were obtained from Lake Van, where the pH is approximately 9. A total of 60 isolates were screened for their potential to produce cellulase. Qualitative cellulase activity assays on CMC-A medium identified 19 cellulase-producing isolates, evidenced by the formation of clear cellulolytic zones around the colonies. Fig S1, provided in Supplementary Materials, highlights the most prominent zones observed in isolates coded V2 (7 mm), V3 (8 mm), V7 (9 mm), VS13 (6 mm), and VS20 (11 mm). The size of the zones provides a preliminary indication of the cellulase production capacity of each isolate. Notably, isolates VS20 and V7 exhibited the largest zones suggesting them promising candidates for further studies.

### Quantitative cellulase activities of the isolates

**Fig. 1:**
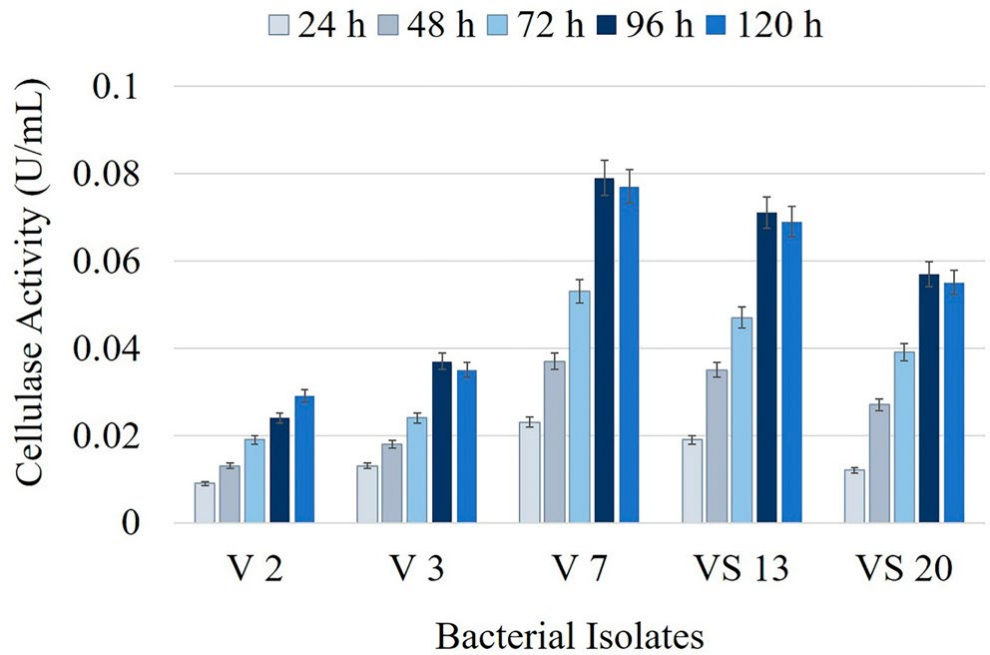
Cellulase activities of the isolates from Lake Van in CMC-B medium during 120-hour incubation at 30 °C

Quantitative activity assays were performed using the five promising bacterial isolates (V2, V3, V7, VS13, and VS20). After incubation in CMC-B medium at 30 °C, 180 rpm for 120 h, cellulase activity was determined using the DNS method, and the isolate exhibiting the highest activity was determined. Based on the zone diameters observed on CMC-A medium and the quantitative cellulase activity results shown in Fig. 1, the bacterial isolate coded V7 was selected as the best cellulase producer.

The carboxymethyl cellulase activity of the V7 isolate was found to be 0.079 U/mL after 96 h of incubation, while its exo-β-1-4 glucanase and β-1-4 glucanase activities were measured as 0.007 U/mL and 0.005 U/mL, respectively. Therefore, in this study, carboxymethyl cellulase activity was primarily emphasized as the key measure of cellulolytic activity.

### Molecular and classical characterization of the most promising cellulolytic isolate

The sequence data based on 16 S rDNA region analysis, were compared with sequences in the NCBI database. The V2 isolate was identified as *Bacillus zhangzhouensis* VLP2 (ON854115.1), the V3 isolate as *B. altitudinis* VLC3 (OR415887.1), the V7 isolate as *B. pumilus* VLC7 (OR415888.1), the VS13 isolate as *B. pumilus* VLCC13 (OR415889.1), and the VS20 isolate as *B. safensis* VLCC20 (OR415890.1). The constructed phylogenetic tree is provided in Supplementary Materials (Fig S2).

Among these isolates, *B. pumilus* VLC7, the best cellulase producer, was further characterized using classical methods. The isolate was motile with peritrichous flagella, rod-shaped, and Gram-positive, with small, cream-colored colonies exhibiting smooth edges. Biochemical tests showed that the isolate was positive for oxidase, hemolysis, catalase, protease, lipase, KOH, asparaginase, maltose, L-arabinose, D-xylose, and myo-inositol, while it tested negative for amylase, glutaminase, and urease. *B. pumilus* VLC7 thrives optimally at 30 °C but can grow across a broad temperature range of 5–45 °C. It also adapts to different salt concentrations, successfully growing in environments with 0–12% NaCl, and shows exceptional pH tolerance, growing well between pH 5 and 11.

### Purification and characterization of cellulase from *B. pumilus* VLC7

In the initial purification step, 250 mL of culture supernatant was precipitated at 40% ammonium sulfate. Buffer A was used to dissolve the precipitate and the sample was subjected to ultrafiltration. After concentration, the sample was then loaded onto a HiTrap Q HP column for further purification. Fractions were collected and assayed for cellulase activity, and those displaying cellulase activity were combined. As shown in Table 1, the 20.5-fold purification process resulted in a 3.7% yield and a specific activity of 16 U/mg.

**Table 1:** Purification steps of *B. pumilus* VLC7 cellulase. Purification was perpormed by ammonium sulphate (40%) precipitation and ion exchange chromatography (HiPrep Q XL 16/10)

Purification Stage	Total volume (mL)	Total protein (mg)	Total activity (U)	Specific activity (U/mg)	Purification Yield (%)	Level of Purification
Supernatant	250	41	32.5	0.79	100	1
Ammonium sulphate precipitation	50	8	19.2	2.4	59	3
Ion exchange chromatography	15	0.075	1.2	16	3.7	20.5

^Specific Activity (U/mg): Cellulase activity (U/mL)/Protein concentration (mg/mL)^

^Yield (%): [Total cellulase activity (U/mL)/Supernatant total activity (U/mL)] x 100^

^Degree of purification: Specific cellulase activity (U/mg)/Supernatant specific protease activity (U/mg)^

**Fig. 2:**
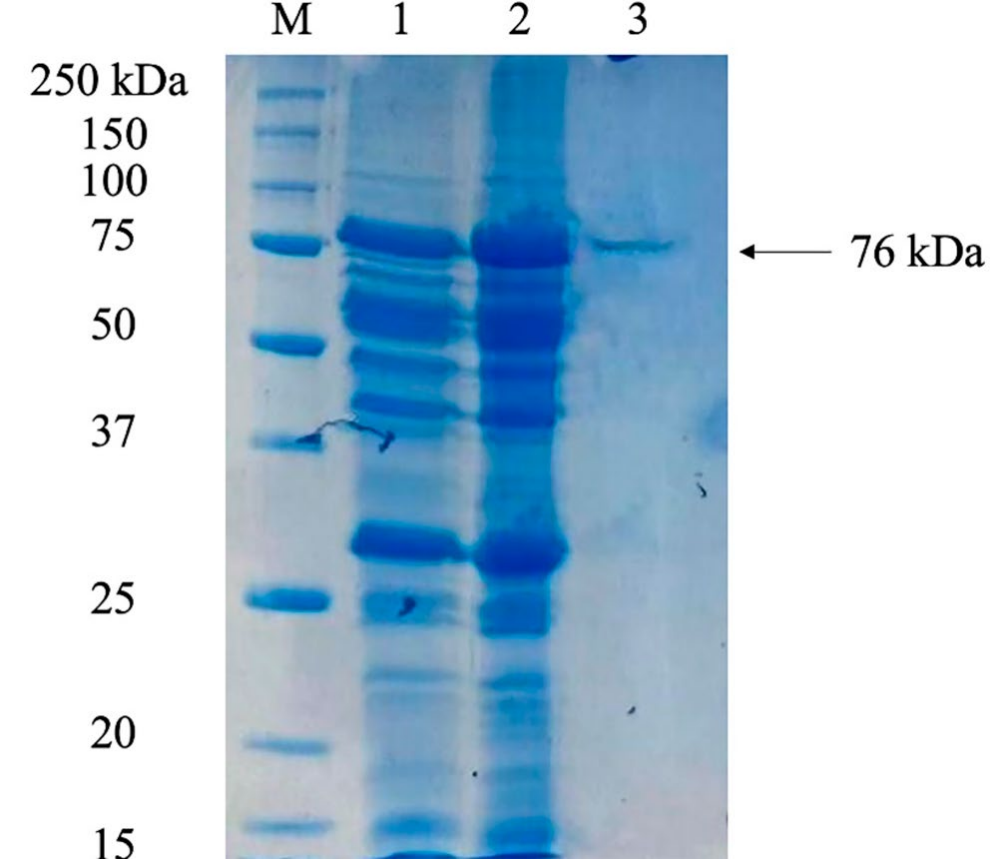
SDS-PAGE of purification samples. M: Molecular weight marker (250–10 kDa); 1: Supernatant; 2: 40% ammonium sulfate precipitation; 3: Ion exchange chromatography

The molecular weight and purity of *B. pumilus* VLC7 cellulase were assessed using SDS-PAGE. Figure 2 shows protein bands from the supernatant (Lane 1) and 40% ammonium sulfate precipitation (Lane 2), with Lane 3 displaying a distinct single band of purified cellulase at an estimated molecular weight of 76 kDa.

The kinetic parameters K_m_ and V_max_ of *B. pumilus* VLC7 cellulase were determined to elucidate its enzymatic efficiency and substrate affinity. The Michaelis-Menten curve along with Lineweaver Burk plot of the cellulase is provided in Supplementary Materials (Fig. S3). For CMC breakdown, the K_m_ and V_max_ values were found to be 0.16 U/mg protein and 3.13 mM, respectively.

### Temperature and pH effects on cellulase activity and stability

**Fig. 3:**
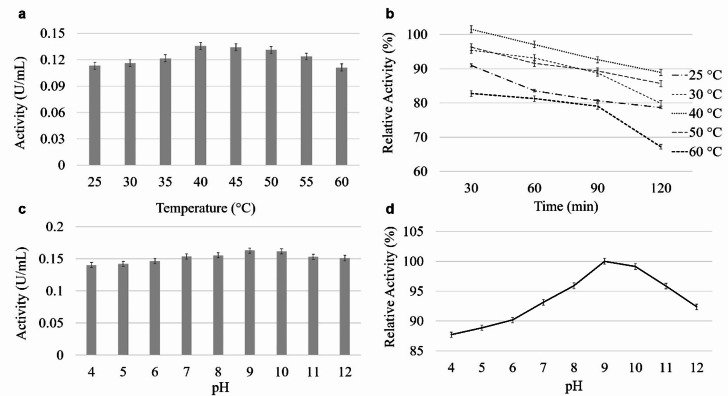
The effects of temperature (**A**, **B**) and pH (**C**, **D**) on *B. pumilus* VLC7 cellulase activity and stability, respectively. The control sample (0.135 U/mL activity for temperature stability and 0.17 U/mL activity for pH stability) was considered to be 100%

The optimal temperature for cellulase activity was found to be 40 °C. However, the enzyme exhibited significant activity at 35 °C and 55 °C, indicating a relatively broad operational temperature range (Fig. 3.a). As for the thermal stability, after 120 min, the enzyme retained 78% of its activity at 25 °C, 79% at 30 °C, 88% at 40 °C, 85% at 50 °C, and 67% at 60 °C (compared to the control sample with 0.135 U/mL activity). Stability was higher at shorter incubation times. At temperatures other than 60 °C, more than 90% of the activity was retained in the first half hour (Fig. 3.b).

The optimum pH for cellulase was found to be 9.0, while the lowest activity was observed at pH 4.0 (Fig. 3.c). In the pH range of 7.0 to 12.0, the enzyme activity remained close to its optimal value. In terms of pH stability, as shown in Fig. 3.d, the enzyme maintained over 95% of its stability in the pH range of 8.0 to 11.0 (compared to the control sample with 0.17 U/mL activity), with pH 9.0 showing particularly high stability.

### Metal ions, inhibitors, and solvents effects on cellulase activity

The effects of metal ions, inhibitors, and solvents on cellulase activity were evaluated by comparing their activity levels to the control sample, which exhibited an activity of 0.151 U/mL and was defined as 100%. Firstly, cellulase activity was evaluated at 5 mM and 10 mM metal ion concentrations. As provided in Supplementary Materials (Fig. S4), MnCl₂ at both 5 mM and 10 mM slightly increased enzyme activity. In contrast, other metal ions reduced cellulase activity to varying degrees, with the greatest reduction (61%) observed at 10 mM HgCl₂, leaving only 39% relative activity.

EDTA and H₂O₂ significantly reduced enzyme activity to 40% and 57%, respectively. On the other hand, SDS and Triton X-100 increased cellulase activity to 127% and 107%, respectively, while Tween 20 (99.8%) and Tween 80 (91%) had little effect on the enzyme. Isopropanol significantly decreased enzyme activity to 40%, while glycerol increased it to 125%. Acetone (76%), ethanol (89%), and DMSO (78%) slightly reduced enzyme activity.

### Impacts of commercial detergents on cellulase activity and stability

**Fig. 4:**
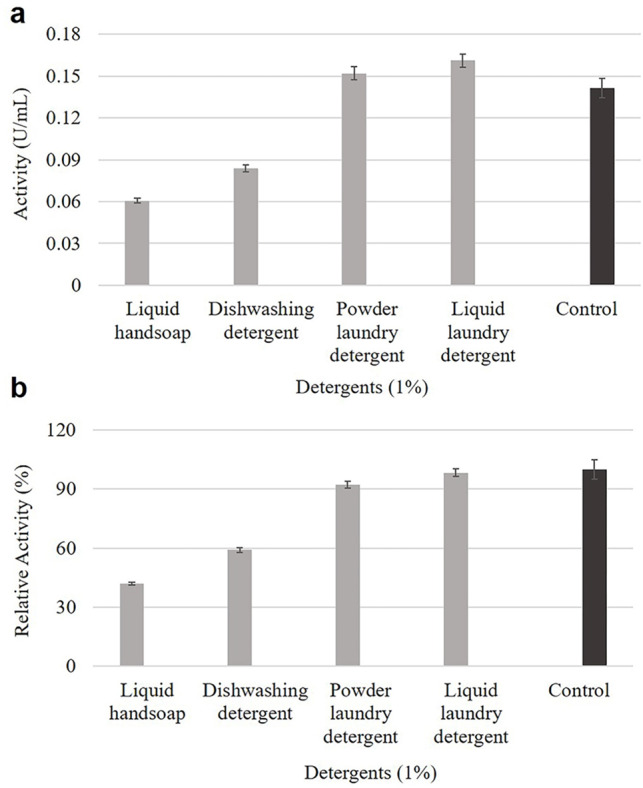
The effects of commercial detergents on *B. pumilus* VLC7 cellulase activity (**A**) and 1-h stability (**B**) in the presence of 1% liquid hand soap, dishwashing detergent, powder and liquid laundry detergents. The control sample with 0.141 U/mL activity was considered to be 100%

Figure 4.a illustrates that cellulase activity enhanced notably in the presence of both liquid laundry detergent (0.161 U/mL) and powdered laundry (0.152 U/mL) detergent when compared to the control (0.141 U/mL), suggesting compatibility with these formulations. Conversely, a reduction in cellulase activity was observed with liquid hand soap and dishwashing detergent. The findings on cellulase activity in the presence of commercial detergents highlight its potential utility in the detergent industry, particularly in laundry formulations.

As demonstrated in Fig. 4.b, cellulase retained a high level of stability in liquid laundry detergent (98%) and powdered laundry detergent (92%) compared to the control sample which exhibited an activity of 0.141 U/mL and was defined as 100%.

### Biotechnological applications of *B. pumilus* VLC7 cellulase

#### Investigation of the bio-polishing effect of cellulase on fabric

**Fig. 5:**
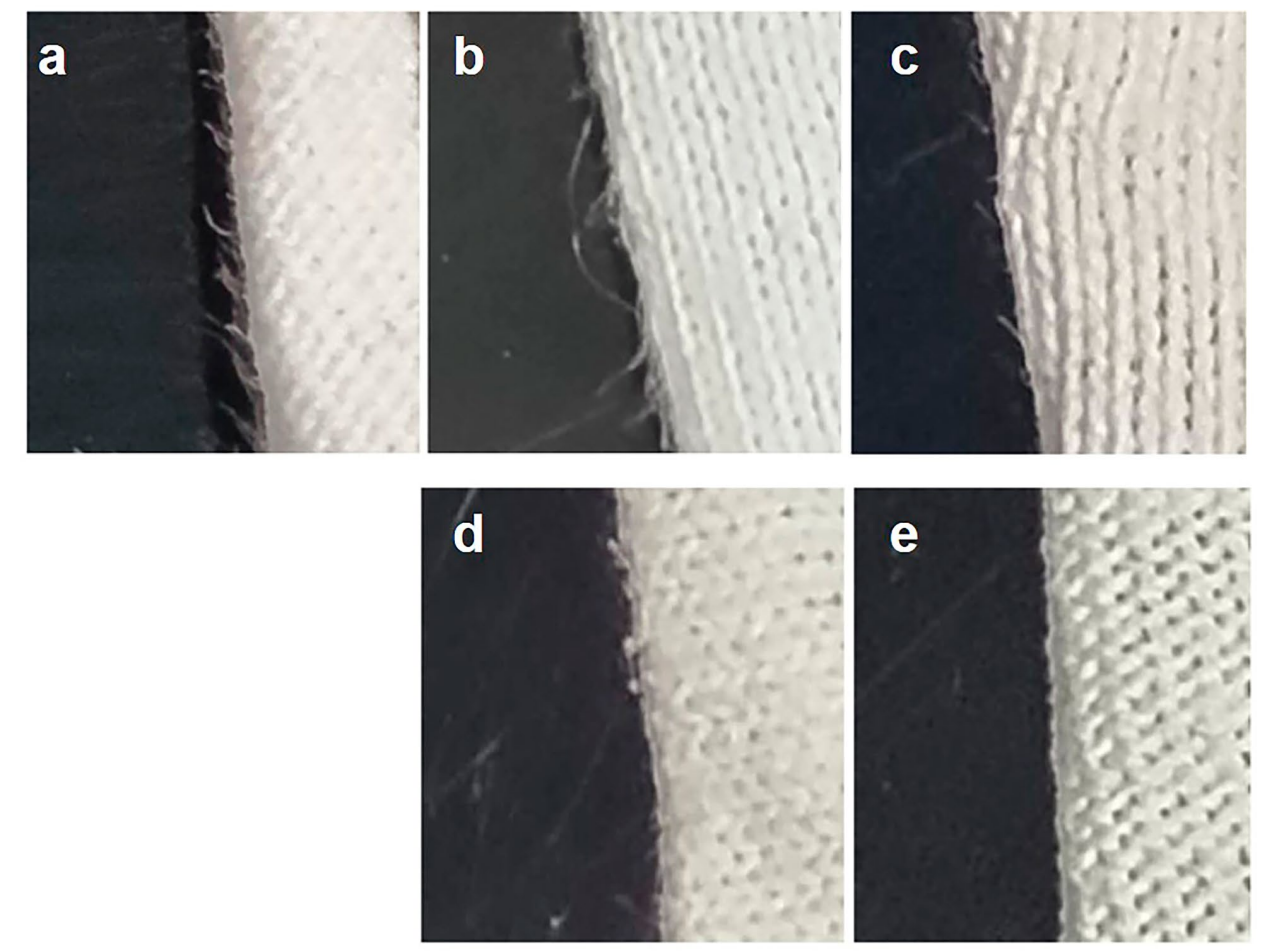
The effect of ***B***. *pumilus* VLC7 cellulase on fabric pilling. **A**: Control (untreated fabric), **B**: After 30 min of incubation in 25 mL, 0.15 U/mL enzyme solution, **C**: 60 min, **D**: 90 min, **E**: 120 min

To evaluate the bio-polishing effect of cellulase on reducing fuzz and pilling on worn fabric surfaces, cotton fabric pieces were incubated in 25 mL culture supernatant containing cellulase (0.15 U/mL) for 15, 30, 45, 60, 90, and 120 min. As shown in Fig. 5, a visible reduction in surface fuzz was observed as early as 30 min, with a progressive increase in fuzz removal over time. By the 120th minute, the surface fuzz had completely disappeared, indicating the enzyme’s effectiveness in bio-polishing.

#### Investigation of the effect of cellulase on fabric dye removal

**Fig. 6:**
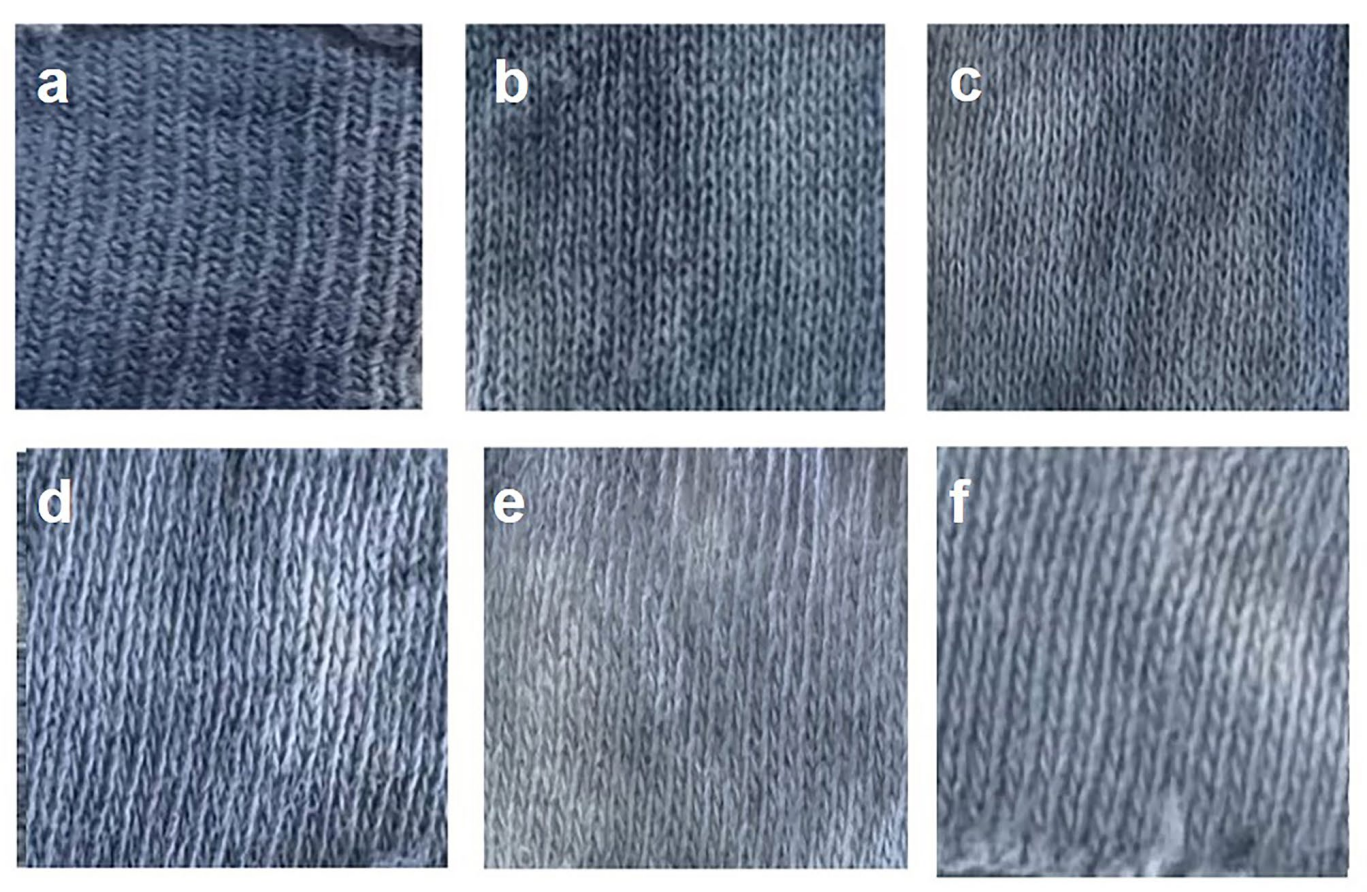
The effect of *B. pumilus* VLC7 cellulase on indigo dye removal from fabric. **A**: Control (untreated fabric), **B**: After 1 h of incubation in 5 mL (0.15 U/mL) enzyme solution, **C**: 3 h, **D**: 5 h, **E**: 15 h, **F**: 20 h

The cotton fabrics stained with indigo dye were incubated in the presence of 5 mL *B. pumilus* VLC7 cellulase solution (0.15 U/mL) for varying durations. As shown in Fig. 6, the longer the incubation time, the greater the visible removal of indigo dye from the fabric. Dye removal was observed even after 1 h, with the most significant removal occurring after 20 h of incubation.

## Discussion

In this study, alkaliphilic bacteria were successfully isolated from Lake Van and screened for their cellulase production potential. Among 60 isolates, five promising strains (V2, V3, V7, VS13, and VS20) demonstrated significant cellulolytic activity, with *B. pumilus* VLC7 (isolate V7) identified as the most efficient producer based on both qualitative and quantitative assays. Molecular and classical characterization confirmed *B. pumilus* VLC7’s identity and revealed its adaptability to diverse environmental conditions, including wide pH and temperature ranges. Lake Van characterized by its extreme alkalinity supports a rich diversity of alkaliphilic microorganisms (Reimer et al. 2009). *Bacillus* species are among the most frequently isolated bacteria from such high pH environments (Ates et al. 2007). As previously indicated bacterial cellulase enzymes are predominantly isolated from *Bacillus* species. *Bacillus* spp. produce three essential cellulases—endoglucanases, exoglucanases, and β-glucosidases—for complete cellulose degradation, with their diverse structures arising from numerous genes; notably, they are also among the few bacteria capable of producing multi-enzyme complexes called cellulosomes (Dobrzyński et al. 2023).

Our study corroborates these findings, as all the most promising alkaliphilic and cellulolytic isolates belonged to the *Bacillus* genus. *Bacillus* species possess specialized mechanisms, such as pH homeostasis and the production of alkali-tolerant enzymes, allowing them to thrive in extreme alkaline conditions. These adaptations make *Bacillus* species particularly valuable for industrial and biotechnological applications, where their resilient enzyme systems are exploited in various processes. Among the isolates, alkaliphilic *B. pumilus* VLC7 demonstrated superior cellulase production and was selected as the source of cellulase enzyme for further study.

*B. pumilus* are widely distributed across diverse habitats and have been commonly isolated from various environments, including deep-sea sediments, soil, and seawater (Pudova et al. 2022; Yakovleva et al. 2022; Zhang et al. 2022). However, this study marks the first time that *B. pumilus* has been isolated from Lake Van for cellulase production. This finding highlights the novelty of this research by expanding the known range of *B. pumilus* to an extreme alkaline environment, underscoring the adaptability and cellulase production of the species.

The cellulase enzyme from *B. pumilus* VLC7 was purified to homogeneity through a multi-step process, achieving a 20.5-fold purification and a specific activity of 16 U/mg. SDS-PAGE analysis confirmed the enzyme’s molecular weight at approximately 76 kDa. These findings highlight the potential of *B. pumilus* VLC7 for industrial cellulase production and its application under challenging conditions. A review of the literature reveals that cellulases from *Bacillus* species commonly have molecular masses ranging from 40 to 80 kDa. Examples include cellulases with molecular weights of 80 kDa from *B. vallismortis* (Gaur and Tiwari 2015) and *Bacillus sp.* HSH-810 (Kim et al. 2005), as well as a 73 kDa cellulase from *B. subtilis* 171ES (Msakni, 2024). However, the molecular weights of cellulases can vary significantly depending on the bacterial species. For instance, a low molecular weight cellulase of 16.9 kDa has been reported from *B. cereus* (Nema et al. 2016). In another study, a mutant *B. pumilus* produced a purified enzyme with a molecular weight of 80 kDa as determined by SDS-PAGE, and 170 kDa when analyzed by gel filtration chromatography (Kotchoni et al. 2006).

The highest activity of *B. pumilus* VLC7 cellulase at pH 9.0 and stability across a broad pH range (7.0 to 12.0) suggests that it is well-suited for alkaline conditions and for use in diverse applications where pH levels may fluctuate, without compromising catalytic performance. In addition, the broad operational temperature range of the enzyme is important for certain applications. In the detergent industry, enzyme stability under alkaline conditions and across a broad temperature range is highly valued (Gurkok 2019). Its thermal stability further enhances its effectiveness in both regular and warm washing cycles, offering versatility across various washing programs. This compatibility with standard detergent formulations supports the inclusion of cellulase in detergent products for improved performance.

The investigation into the effects of metal ions, reagents, and organic solvents on cellulase activity provides valuable insights for optimizing enzyme use in industrial applications. The slight increase in *B. pumilus* VLC7 cellulase activity with Mn²⁺ suggests its potential as a cofactor, possibly improving enzyme-substrate interactions or stabilizing the enzyme’s active conformation. In contrast, Hg²⁺ ions strongly inhibited cellulase activity, likely due to their interaction with sulfhydryl groups or other critical enzyme residues, leading to structural disruptions (Tejirian and Xu 2010). For industrial processes, avoiding heavy metal contamination or employing chelating agents may help maintain cellulase efficiency. To mitigate such inhibition, strategies such as enzyme modification or the use of metal ion chelators could be explored to shield cellulase from metal-induced deactivation. Additionally, the data suggest that optimizing the metal ion composition in reaction mixtures can enhance the efficiency of cellulase in industrial applications. For instance, the inclusion of Mn²⁺ in cellulase formulations could improve catalytic performance under specific conditions, opening avenues for more effective enzyme-based processes.

SDS and Triton X-100, both surfactants, significantly enhanced cellulase activity, suggesting that they may help improve substrate accessibility by altering the cellulose surface or disrupting substrate aggregates. These reagents could be useful in processes where cellulase is used to break down complex or hydrophobic substrates. However, EDTA, a chelating agent, and H₂O₂, an oxidizing agent, reduced activity, indicating that metal ion chelation or oxidative damage to the enzyme could compromise its function.

Glycerol’s enhancement of cellulase activity (125%) suggests it might stabilize the enzyme’s structure, reducing denaturation and improving activity. The enhancement of enzyme activity by organic solvents may be due to the nonpolar, hydrophobic solvent residues providing an interface that keeps the enzyme in an open structure, leading to stimulated activation (Zaks and Klibanov 1988). However, solvents like isopropanol significantly reduced cellulase activity, possibly due to solvent-induced protein denaturation or interference with the enzyme’s active site. Solvent-tolerant enzymes as well as enzymes inhibited by solvents such as ethanol have been previously reported (Annamalai et al. 2013; Gaur and Tiwari 2015). The activity of cellulase produced from *B. pumilus* VLC7 strain increased in the presence of SDS and Triton X-100, while it decreased in the presence of H_2_O_2_ and EDTA. Similar results have been shown previously with different *Bacillus* cellulases (Trivedi et al. 2011; Asha and Sakthivel 2014).

*B. pumilus* VLC7 cellulase activity increased significantly in the presence of both liquid and powdered laundry detergents. These results underscore the enzyme’s potential for applications in the detergent industry, particularly in enhancing the performance of laundry formulations. Cellulase demonstrated remarkable stability in both liquid and powdered laundry detergents. This stability is essential for its use in cleaning products, as the enzyme must remain active during the washing process to be effective and suggests that cellulase could be integrated into both liquid and powdered laundry detergents for improved stain removal performance and bio-polishing effect. In contrast, the enzyme’s stability dropped markedly in the presence of dishwashing detergent (59%) and liquid hand soap (42%), likely due to interactions with certain surfactants or other inhibitory agents present in these detergents. Surfactants or other chemicals might inhibit the enzyme by altering its structure or blocking its active sites. This suggests that cellulase might be less suited for these applications unless the formulation is specifically tailored to protect the enzyme. Unlike the present study, many studies in the literature report a much greater decrease in cellulase activity in the presence of commercial detergents (Gaur and Tiwari 2015; Annamalai et al. 2013).

The bio-polishing effect of *B. pumilus* VLC7 cellulase on cotton fabric surfaces led to a significant reduction in surface fuzz within a short period. Most detergent manufacturers use various cellulase blends to maintain fabric care and preserve the whiteness or color clarity of textiles. After several washes, microfibrils form on the cotton fibers of garments made from cotton or cotton blends. These fibers gradually lose their smooth appearance and begin to look fuzzy. The microfibrils scatter light, turning whites to a grayish shade and making bright colors appear dull and muted. Cellulases remove these protruding fibers (fuzz) from cotton and other cellulose components in synthetic fibers, smoothing the fabric and enhancing its brightness (Breen and Singleton 1999). They also remove solid dirt, preventing the reaccumulation of stains and dust particles (Rähse 2014). Additionally, cellulases improve detergent effectiveness by breaking down microfibrils within particulate soils like ink or mud (Sukumaran et al. 2005). Cellulase enzymes are also used to enhance fabric elasticity, softness, and resistance to washing (Gurkok 2019).

The study on the bio-polishing effect of *B. pumilus* VLC7 cellulase highlights the enzyme’s potential in textile finishing processes. The progressive reduction in surface fuzz observed on the fabric pieces after cellulase treatment suggests that the enzyme effectively hydrolyzes exposed cellulose fibers, leading to a smoother fabric surface. This bio-polishing action could be a sustainable alternative to mechanical finishing techniques, reducing fabric pilling and enhancing fabric quality without the need for harsh chemicals or abrasive processes. The complete removal of fuzz after 120 min underscores the enzyme’s efficiency and applicability for commercial textile treatment.

The investigation into the dye removal effect of *B. pumilus* VLC7 cellulase demonstrates the enzyme’s potential in textile dyeing and washing processes. The enzyme was capable of removing indigo dye from cotton fabric, with the degree of removal increasing with longer incubation times. This suggests that cellulase can break down dye-fiber bonds, particularly under specific conditions like prolonged exposure, making it a useful tool in fabric bio-bleaching and eco-friendly textile recycling processes. The ability to achieve significant dye removal within a relatively short time (1 h) enhances its attractiveness for industrial applications.

In previous studies, the usability of cellulase enzymes of *Bacillus* strains in the detergent industry has been investigated and their potential to remove impurities from cotton fabrics has been studied and suggested to be suitable for this purpose (Ito 1997; Hoshino et al. 2000). The present study highlights the versatility of cellulase in the textile industry, offering eco-friendly alternatives for fabric treatment, bio-polishing, and dye removal, while potentially improving sustainability and reducing environmental impact.

## Conclusion

The purified cellulase enzyme from *B. pumilus* VLC7 exhibits promising potential for various industrial applications, particularly in the textile and detergent industries. The enzyme displayed an optimum temperature of 40 °C, with significant activity observed at temperatures of 35 °C and 55 °C, indicating a broad operational temperature range. It also demonstrated an optimal pH of 9, with high stability in the alkaline range, making it suitable for applications in high-pH environments, such as detergents.

When tested with various detergents, the cellulase retained a large portion of its activity in liquid and powdered laundry detergents, showcasing compatibility with detergent formulations. Additionally, the enzyme showed a notable bio-polishing effect on cotton fabric, effectively reducing fuzz and pilling after 30 min of incubation, with complete removal achieved after 120 min. This indicates its potential use in the textile industry for improving fabric quality. The cellulase also demonstrated the ability to remove indigo dye from fabric, with the most significant dye removal occurring after 20 h of incubation, suggesting a potential application in fabric dyeing and recycling processes.

In conclusion, the cellulase derived from *B. pumilus* VLC7 demonstrates notable potential in bio-polishing, dye removal, and detergent formulations, providing an eco-friendly and versatile option for enhancing fabric quality and detergent performance in both industrial and household settings. Its stability in alkaline conditions and broad operational temperature range make it particularly suitable for sustainable industrial processes. The findings of this study lay a strong foundation for further exploration of the biotechnological applications of cellulase enzymes, especially in addressing challenges in sustainability. Given its alkaline stability and broad temperature range, this cellulase could be integrated into eco-friendly processes, reducing the reliance on harsh chemicals and minimizing environmental impact.

## Electronic supplementary material

Below is the link to the electronic supplementary material.

Supplementary Material 1

## Data Availability

No datasets were generated or analysed during the current study.
