# Supplementary material for: Biotechnological uses of purified and characterized alkaline cellulase from extremophilic Bacillus pumilus VLC7 from Lake Van
Source: World J Microbiol Biotechnol. 2025 Feb 4;41(2):60. doi: 10.1007/s11274-025-04271-4 (PMC11790762; doi:10.1007/s11274-025-04271-4)
Supplement: Supplementary file 1 — Supplementary Material 1 [file 11274_2025_4271_MOESM1_ESM.pdf]

World Journal of Microbiology and Biotechnology

Biotechnological Uses of Purified and Characterized Alkaline Cellulase from Extremophilic *Bacillus pumilus* VLC7 from Lake Van

Aysun Ayse Yilmaz[a], Sumeyra Gurkok[b*]

[a]Institute of Natural and Applied Sciences, Atatürk University, Erzurum, Turkey, ORCID: 0000-0001-9071-7897

[b]Department of Biology, Science Faculty, Ataturk University, Erzurum, Turkey, ORCID: 0000-0002-2707-4371

Corresponding author: Sumeyra Gurkok, E-mail: sumeyrag@gmail.com, sgurkok@atauni.edu.tr

Supplementary Materials

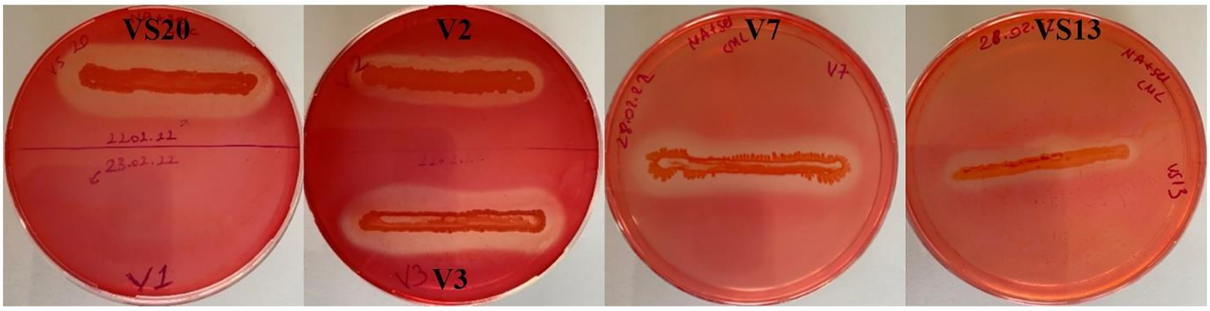

Fig. S1 Cellulolytic zones formed by bacterial isolates from Lake Van after incubation at 30 °C for 48 hours on CMC-A medium.

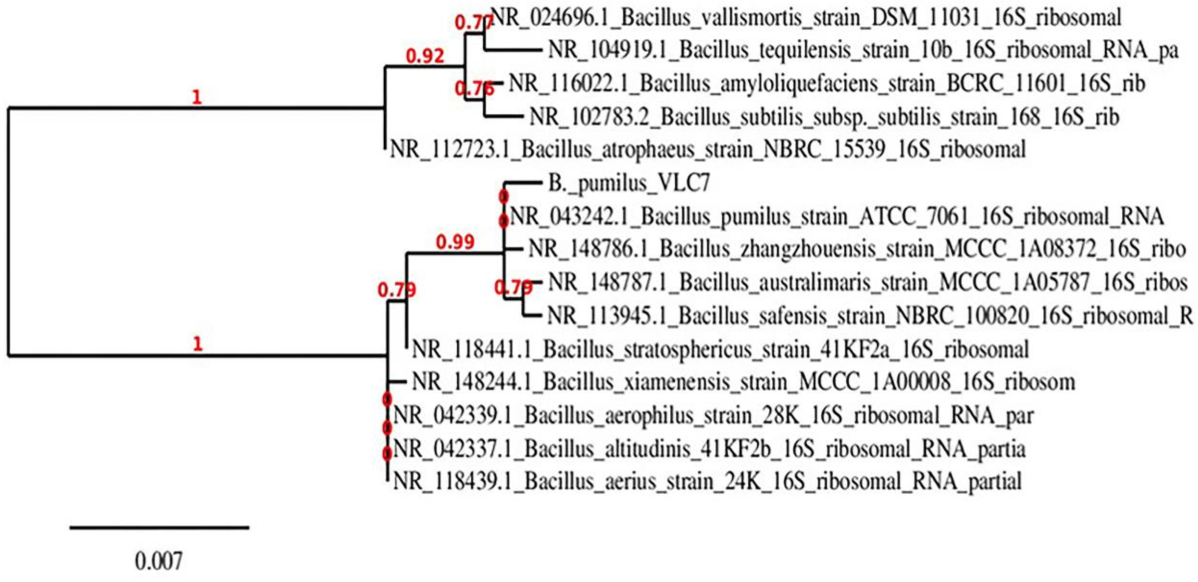

Fig. S2 Phylogenetic tree for B. pumilus VLC7 constructed by Maximum-likelihood analysis of 16S rRNA gene sequences

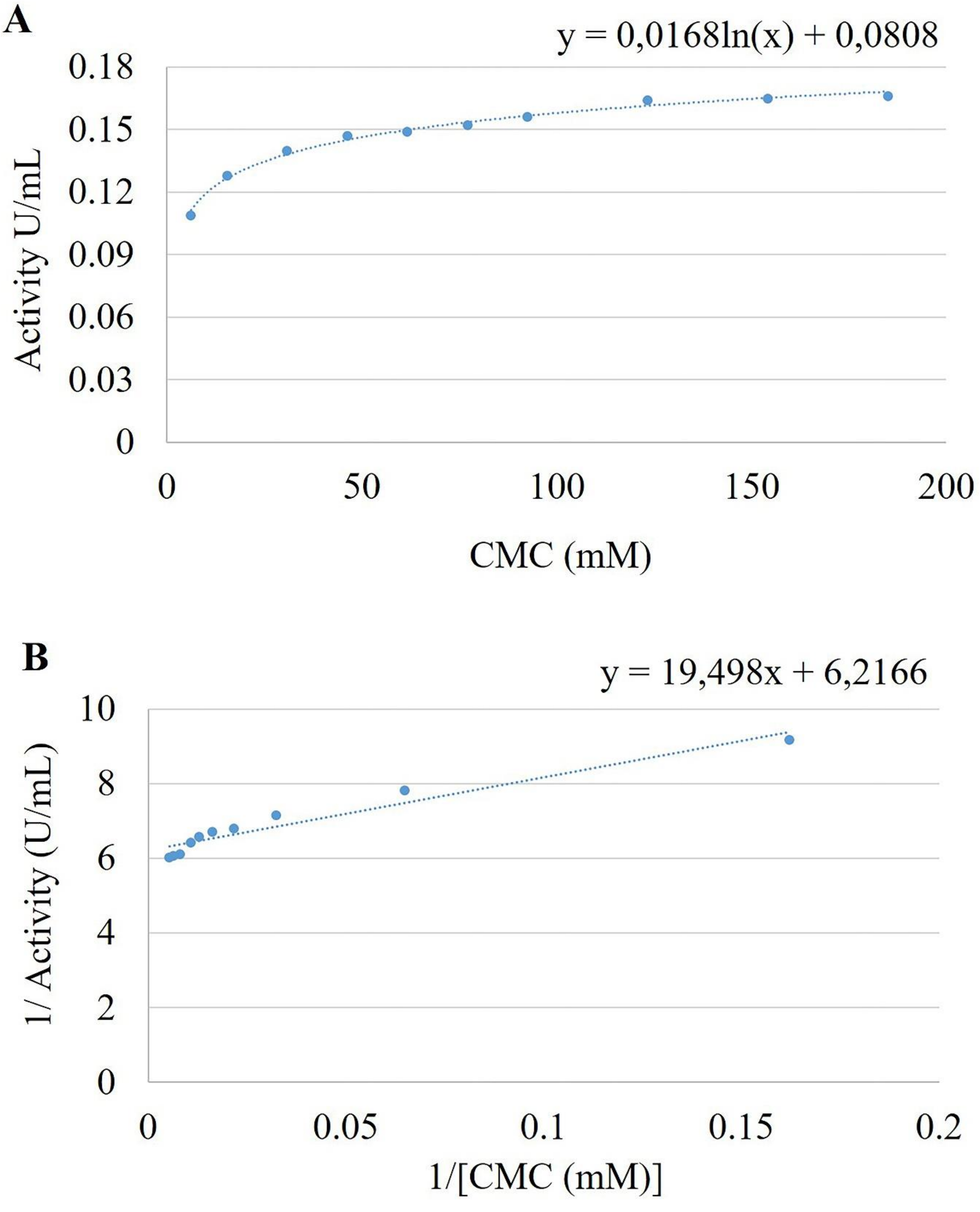

Fig. S3 Michaelis-Menten plot (A) and Lineweaver-Burk plot (B) of *B. pumilus* VLC7 cellulase

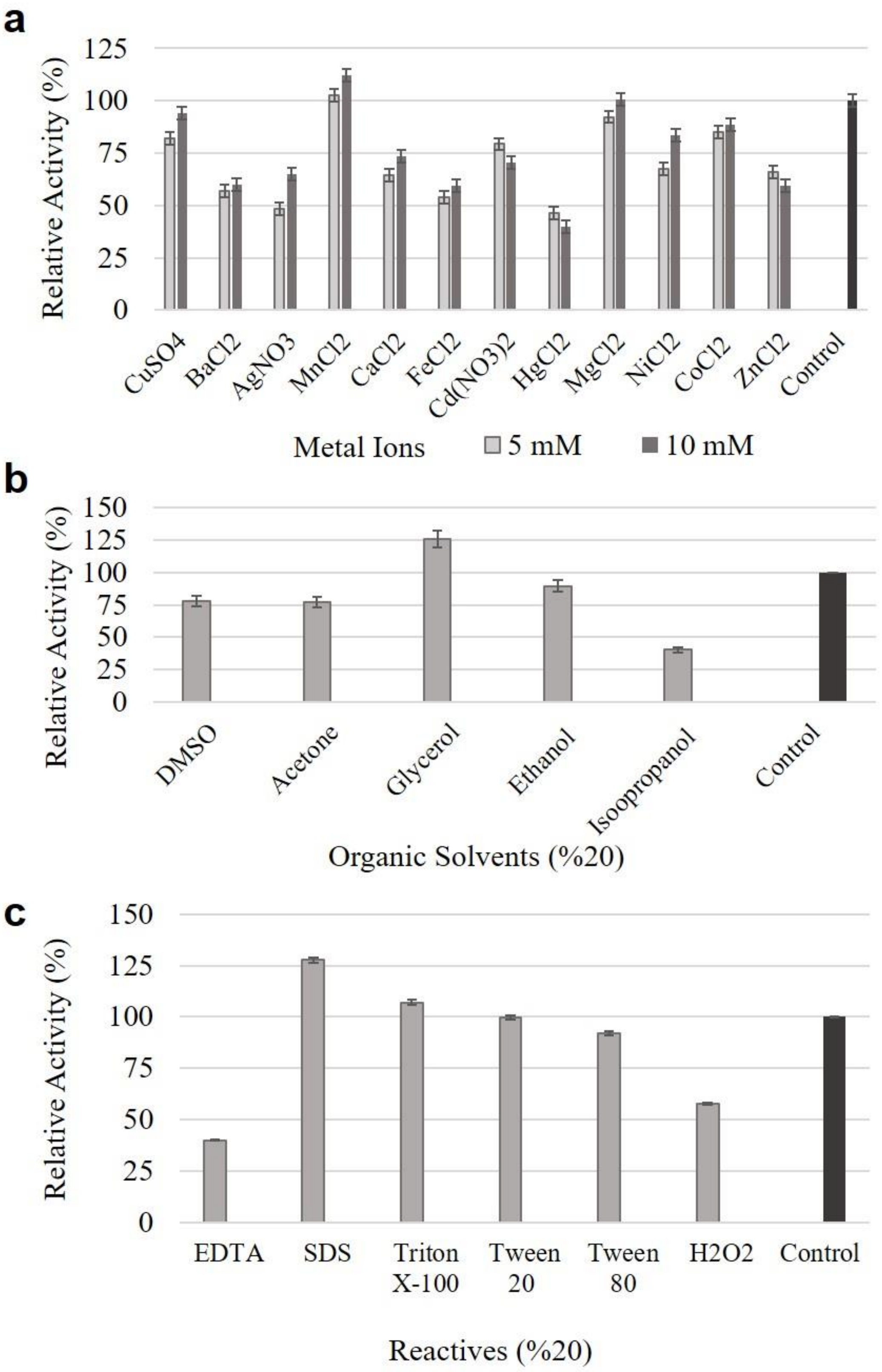

Fig. S4 The effects of metal ions (a), organic solvents (b), and reactives (c) on *B. pumilus* VLC7 cellulase. The control sample with 0.151 U/mL activity was considered to be 100%
